# A working method for estimating dynamic shear velocity in the montney formation

**DOI:** 10.1016/j.mex.2019.08.013

**Published:** 2019-08-23

**Authors:** Sochi C. Iwuoha, Per K. Pedersen, Christopher R. Clarkson, Ian D. Gates

**Affiliations:** a Department of Geosciences, University of Calgary, Canada; b Department of Chemical and Petroleum Engineering, University of Calgary, Canada

**Keywords:** DTP, sonic log – compressional slowness, DTS, sonic log – shear slowness, NNE, neural network estimation, GR, gamma ray log, RCW, reservoir characterization workflow, RHOB, bulk density, SSTVD, subsea true vertical depth in meters, Vp, compressional sonic velocity, Vs, shear sonic velocity, Vs_ANN_, shear velocity log estimated using artificial neural network techniques, Vs DOL, shear velocity log estimated using Greenberg-Castagna [1] Dolomite lithology constants, Vs SH, shear velocity log estimated using Greenberg-Castagna [1] Shale lithology constants, Vs SST, shear velocity log estimated using Greenberg-Castagna [1] Sandstone lithology constants, Vs LST, shear velocity log estimated using Greenberg-Castagna [1] Limestone lithology constants, Vs MDRK, shear velocity log estimated using Castagna et al. [5] Mudrock lithology constants, Vs MJ Clavier, shear velocity log estimated using Marion & Jizba [11] method with Clavier et al. [12] fractional clay volume correction, Vs MJ Larionov, shear velocity log estimated using Marion & Jizba [11] method with Larionov [13] fractional clay volume correction, Vs MJ Stieber, shear velocity log estimated using Marion & Jizba [11] method with Stieber [14] fractional clay volume correction, Vs_Regress_, shear velocity log estimated from the bivariate analysis of dipole sonic Vp and Vs logs, V*sh, Clavier*, Clavier et al. [12] fractional clay volume correction, V*sh, Larionov*, Larionov [13] fractional clay volume correction, V*sh, Stieber*, Stieber [14] fractional clay volume correction, Dynamic shear velocity estimation from compressional velocity logs in the Montney Formation, Log analysis, Velocity, Correlation, Tight reservoir, Siltstone, Shale

## Abstract

In this paper, we present a customized method for estimating sonic shear velocity (Vs) from compressional velocity (Vp) logs in the Montney Formation, in wells lacking dipole sonic data. Following a multi-scenario analysis that comprised of assessing empirical Vs estimation relations [including lithology, porosity (Ø), and volume of clay (V_sh_)-based Vs estimation techniques], bivariate statistics, and machine learning, we found that the Greenberg & Castagna (1992) shale lithology constants yield Vs log estimates that best match the measured Montney Formation Vs in our study area, with a regional correlation coefficient of 0.8. We have therefore customized the Vs estimation method in our study to use the Greenberg & Castagna (1992) shale lithology constants. Our working method:

• Improves the efficacy of Vs log estimation from Vp logs in the study area
• Demonstrates the importance of calibrating empirical relations for Vs estimation to a specific formation, and
• Provides a more accurate complementary Vs log dataset for subsequent regional reservoir characterization studies

## Graphical abstract

**Figure:**
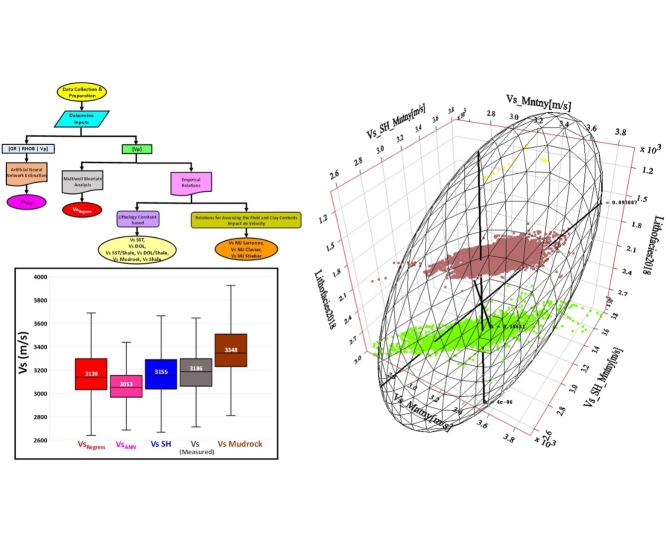

**Specifications Table**

**Table:** 

Subject Area:	Earth and Planetary Sciences
More specific subject area:	Well log analysis
Method name:	Dynamic shear velocity estimation from compressional velocity logs in the Montney Formation
Name and reference of the original method:	**Method 1**: Shear velocity (Vs) estimation from compressional velocity (Vp) logs using sandstone, dolomite, and shale lithology constants. Developed by Greenberg-Castagna [1]. **Method 2**: Shear velocity (Vs) estimation from compressional velocity (Vp) logs using mudrock lithology constants. Developed by Castagna et al. [5] The Greenberg-Castagna [1] and Castagna et al. [5] methods were developed based on a combination of laboratory and field tests that considered the velocities for porous water-saturated pure lithologies, mixing laws for solid rock constituents and the application of the Biot-Gassmann theory to real rocks. **Method 3**: Vs estimation based on porosity (Ø) and clay volume (V_sh_). Developed by Marion & Jizba [11]. This method is based on laboratory acoustic and ultrasonic measurements. **Method 4**: Bivariate statistics: Analyzing two variables (Vp and Vs for this study) to identify the relationship between both variable to predict one variable from the other [25]. **Method 5**: Artificial Neural Network Analysis [22]. Applying Methods 1-3 above for Vs estimation requires calibration for specific geologic formations, hence the customized working method for estimating and validating Vs logs as will be presented in this paper. Methods 4 and 5 served as complementary techniques tested to confirm that the working method we have customized is the technique that yields the most optimal Vs estimates in the study area.
Resource availability:	The method proposed in this paper can be reproduced in commercial industry software such as Petrel, GOCAD, GeoScout, Techlog, or Interactive Petrophysics (to mention but a few), as well as in Microsoft Excel.

## Method details

### Area of study and geological setting

**Fig. 1:**
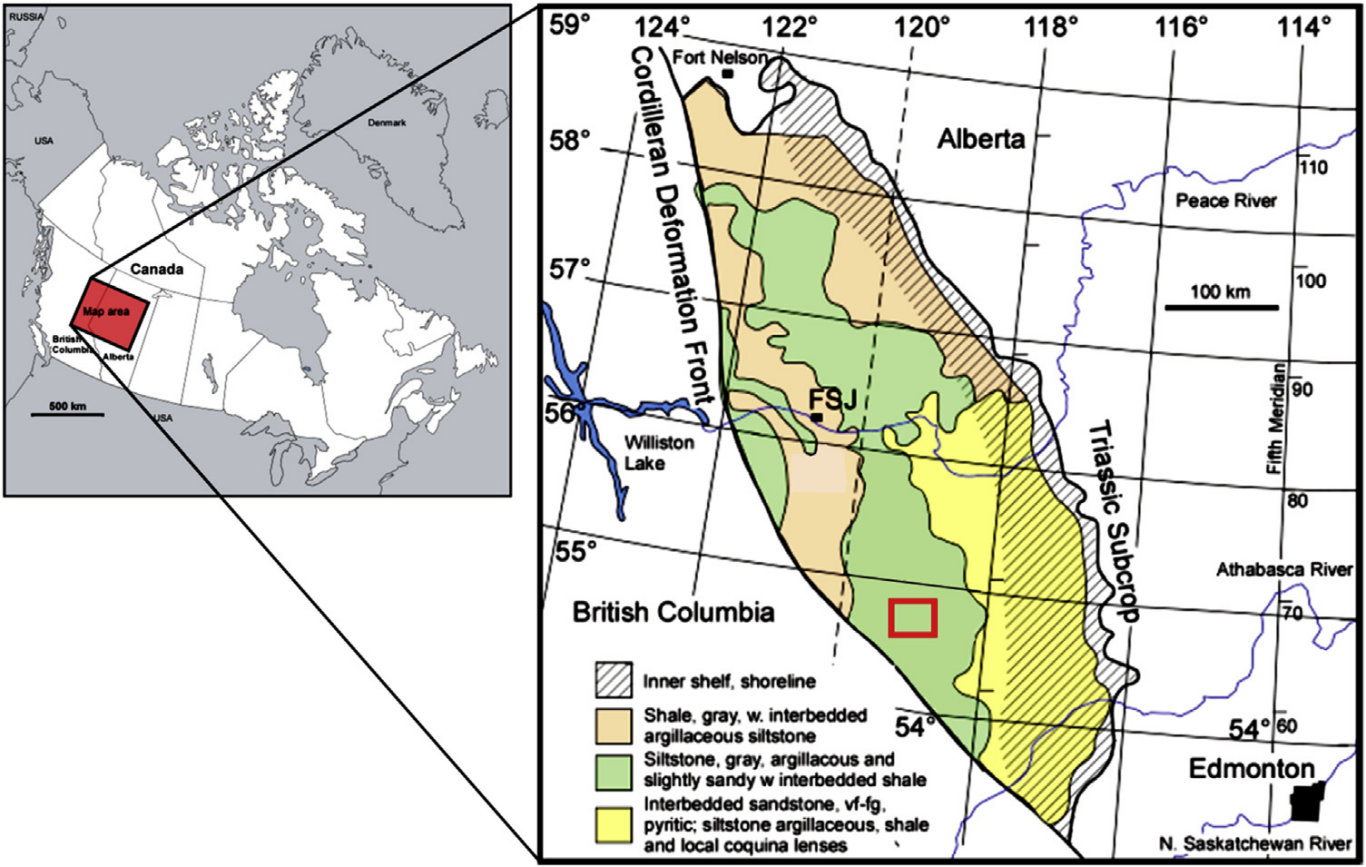
Study area. The rectangle represents the area of interest in this study. Modified from [16].

**Fig. 2:**
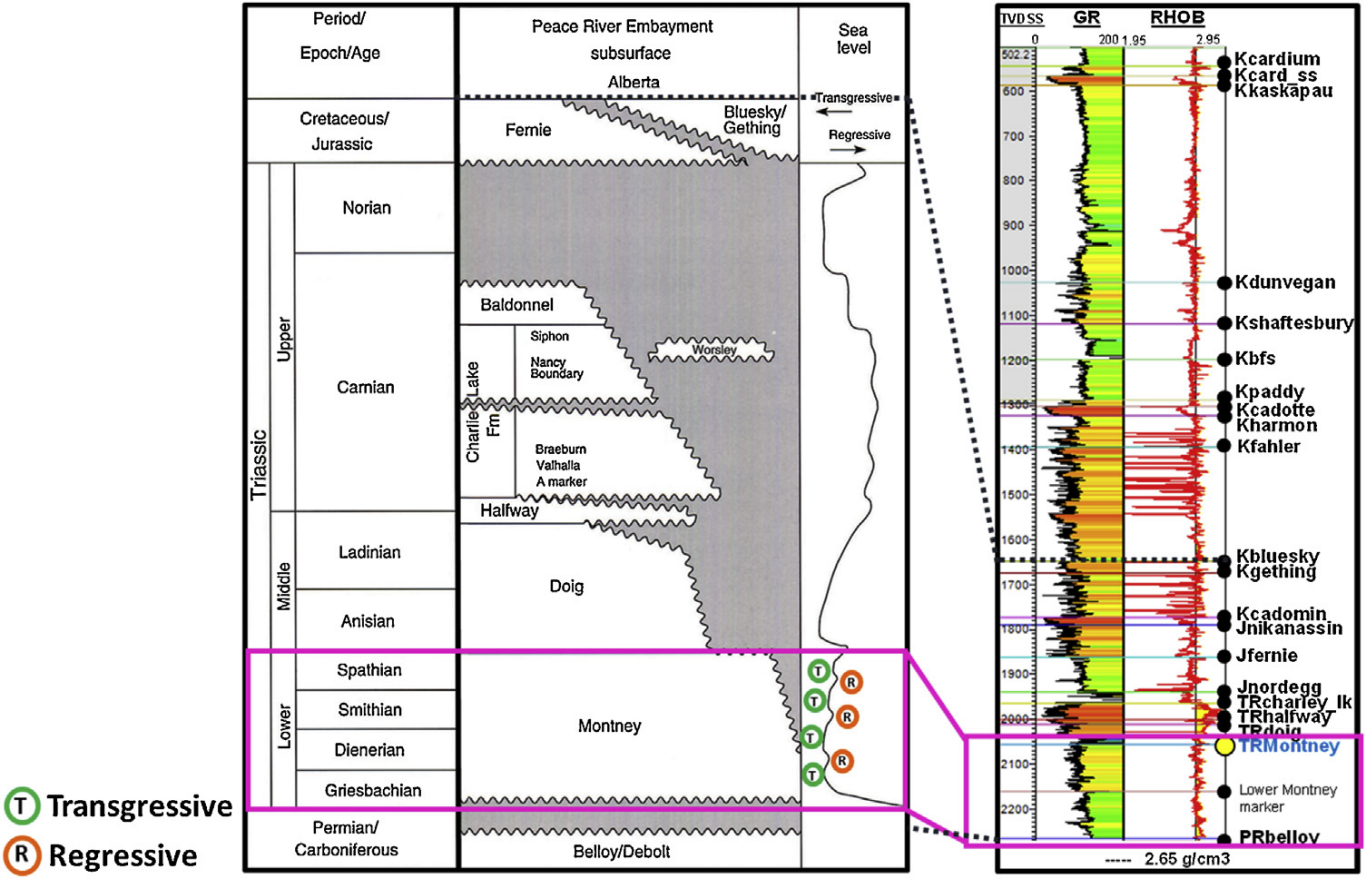
Stratigraphic section (Triassic to Cretaceous interval) from a type well in the study area, compared to the Peace River embayment subsurface succession in Alberta. Modified from [15] and [10].

The study area covers 1182 km^2^ (˜292,000 acres) in the eastern Peace River Arch area in west-central Alberta, Canada (Fig. 1). The geologic interval of interest is the Lower Triassic Montney Formation. The Montney Formation is a west-dipping clastic wedge that ranges from shallow-water interbedded sands, silts and shales in the east to deep-water shales with interbedded argillaceous siltstones in the west (Fig. 1). The Montney Formation lithology is the area of this study is comprised of siltstones and sands with interbedded shales (Fig. 1, Fig. 2). The sandy portions in the study area have been described in the literature as being comprised of fine silty dolomitic sandstone [17].

**Fig. 3:**
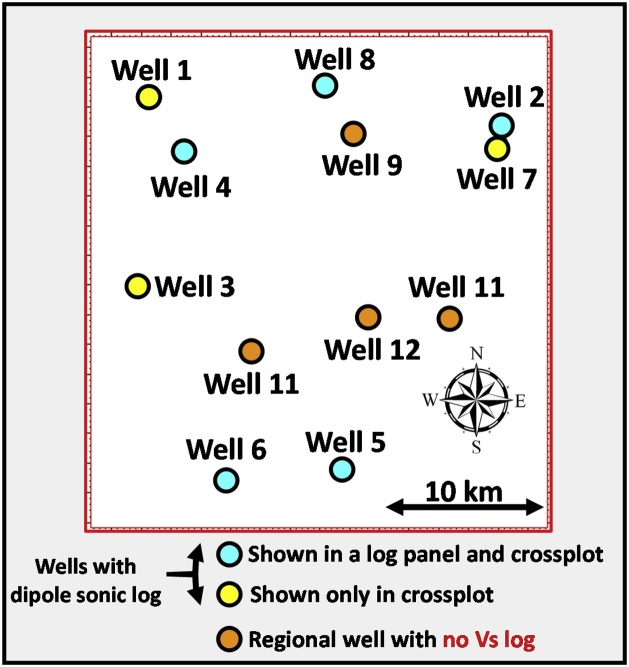
Well layout of the study wells.

### Objective and background

The purpose of the working method presented in this paper was to estimate shear velocity (Vs) logs in vertical wells where only compressional velocity (Vp) sonic log data was available. The estimated Vs logs were to be incorporated into a reservoir characterization workflow (RCW) [2] that was being developed to use Vs, Vp, and bulk density (RHOB) logs to map regional petrophysical and geomechanical properties in the Montney Formation in the study area (Fig. 1**)**.

Twelve vertical wells were available for the regional RCW being developed [2], however, dipole sonic logs were only available in eight of the twelve wells. To improve the spatial calibration of the Vs data over the study area, thus enhancing the predictive capability of the regional RCW in interwell areas, it was necessary to utilize a complete set of Vs logs from the twelve vertical wells.

At the time of this study, we were unaware of any Montney Siltstone-calibrated theoretical relation for estimating dynamic Vs from Vp logs that could be applied to the vertical wells in our study area. Previously, efforts had been made to establish a relationship between static (core/laboratory-based) and dynamic (well log-based) Vs and Vp [3,4] in the Montney Formation. The studies could inform static to dynamic Vp and Vs estimations (where core data are available), however, these studies were based on core data in a limited area and did not assess the potential for estimating or calibrating Vs logs from Vp logs at a regional scale. Therefore, we customized the method presented in this paper for testing and validating Vs logs estimated from Vp logs in our Montney study area, as will be presented below.

### Method and validation

**Fig. 4:**
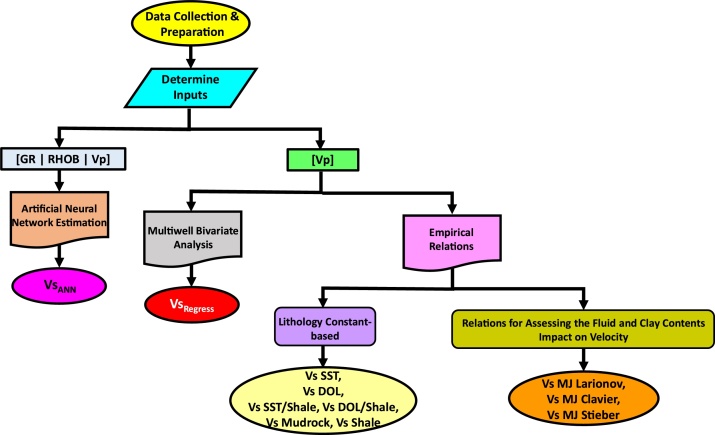
Workflow to determine the most optimal Vs log estimation approach for the Lower Triassic Montney Formation.

The customization workflow involved evaluating different methods of estimating Vs from Vp logs (Fig. 4). We investigated the use of empirical relations (Fig. 4) because in many cases, they are the default methods provided for use in industry software but in many cases may not have been calibrated for the geologic formation within which a Vs estimation is being performed (as is our case in this study).

A complementary multiwell bivariate analysis was conducted to determine the predictive capability when a regional Vp – Vs log relationship is used (rather than empirical relations) to generate Vs logs in the study area. Furthermore, machine learning (artificial neural network estimation) was used to compare the predictive capability when the Vp log is combined with other logs to estimate Vs in the study area.

As is indicated in the workflow (Fig. 4), prior to commencing the data analysis, the input data were collated and prepared. The gamma-ray (GR), bulk density (RHOB), compressional and shear sonic transit time (DTP and DTS) logs from eight vertical wells were imported and quality-controlled for outlier values. Following the quality control, the DTP and DTS logs were converted into Vp and Vs logs using the relations below:

(1) $$Vp=\frac{1}{DTP}$$

(2) $$Vs=\frac{1}{DTS}$$

Where: Vp is Compressional velocity (m/s), Vs is Shear velocity (m/s), DTP is compressional sonic transit time (s/m), and DTS is shear sonic transit time (s/m).

### Empirical methods for vs estimation from Vp logs

To estimate Vs from Vp logs, we utilized the theoretical relations developed by Greenberg and Castagna [1] which estimate Vs from Vp logs using lithological constants for sandstone, limestone, dolomite, and shale. These relations are expressed as:

(3) Vs SST=0.8042Vp-855.9

(4) $$Vs\;LST=1.0168Vp-0.00005509{Vp}^{2}-1030.5$$

(5) Vs DOL=0.5832Vp-77.76

(6) Vs SH=0.77Vp-867.4

Where: Vs SST is Vs (m/s) estimated using Sandstone lithology constants, Vs LST is Vs (m/s) estimated using Limestone lithology constants, Vs DOL is Vs (m/s) estimated using Dolomite lithology constants, and Vs SH is Vs (m/s) estimated using Shale lithology constants.

Furthermore, we calculated Vs from the Vp logs of the four wells using the Castagna et al. relation [5] for Vs estimation in mudrocks. Castagna et al. [5] defined mudrocks as clastic silicate rocks that are mainly composed of clay and silt-sized particles. Their relation is given as:

(7) $$Vs\;Mudrock=\frac{Vp\;-\;1360}{1.16}$$

Where: Vs Mudrock is Vs (m/s) estimated using mudrock lithology constants

**Fig. 5:**
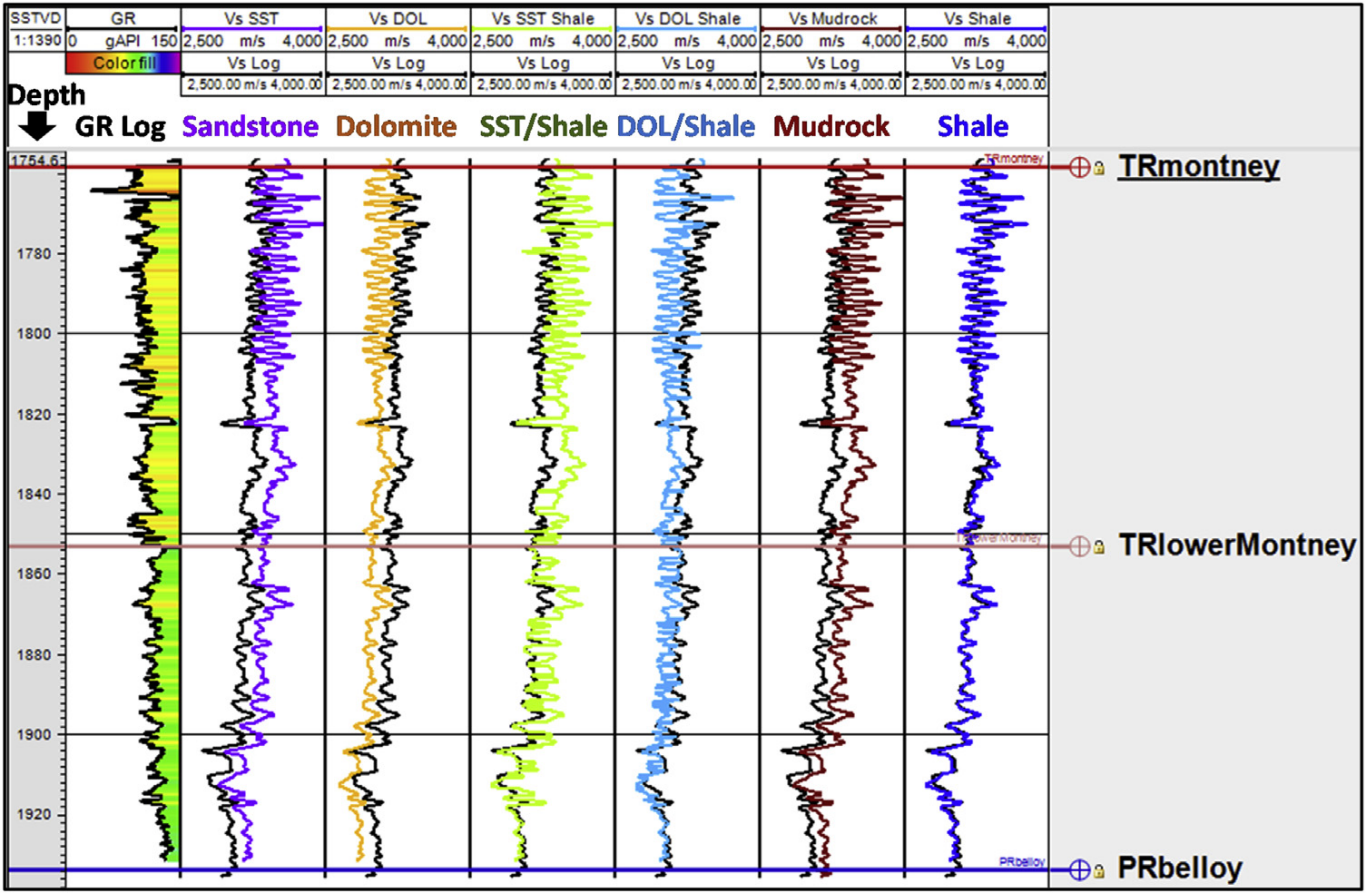
Lithology constant-based scenarios for estimating Montney Vs from Vp log in well 2. The depth scale is in meters subsea true vertical depth (mSSTVD). The first two letters of the formation tops refer to the geologic age. TR is Triassic and PR is Permian.

**Fig. 6:**
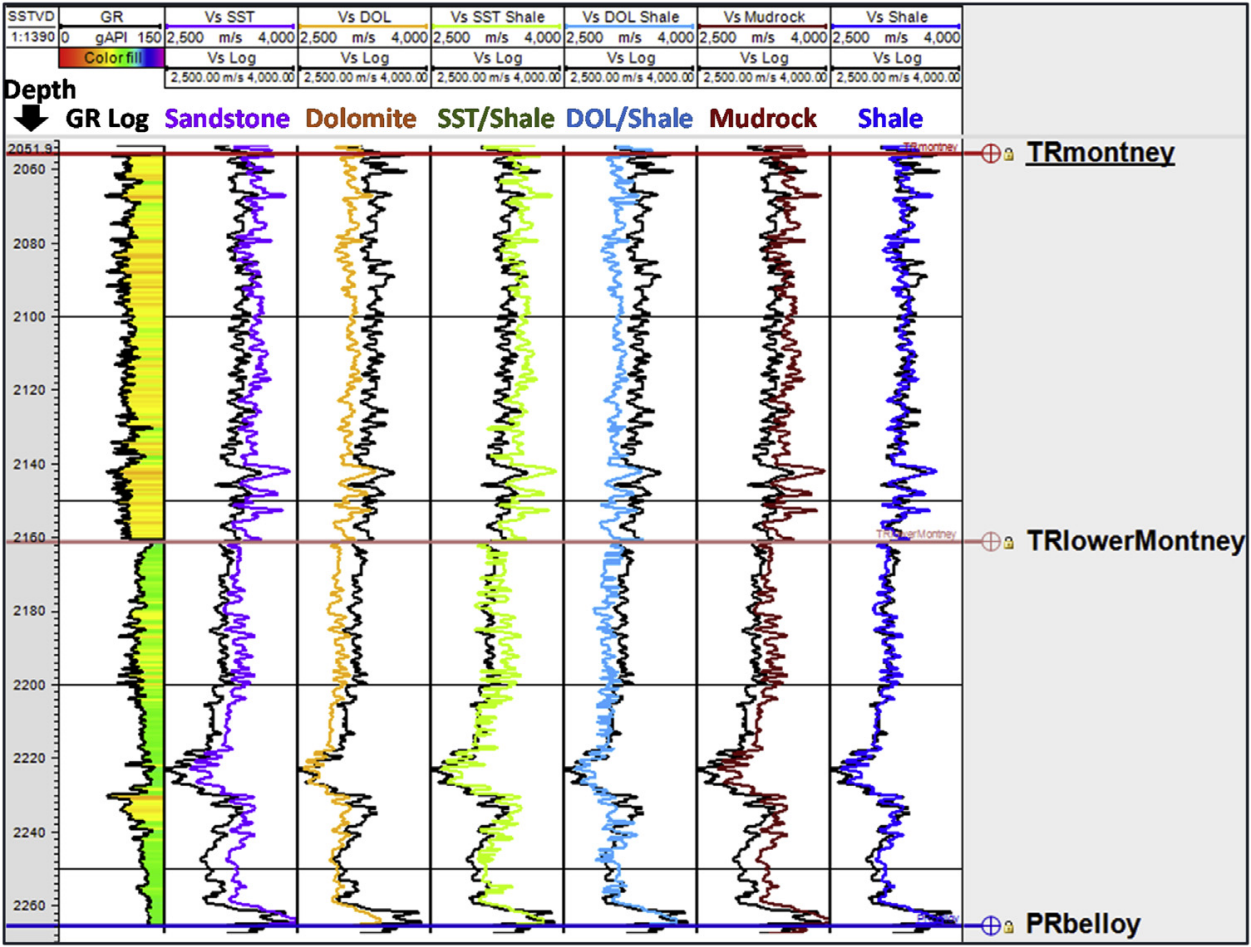
Lithology constant-based scenarios for estimating Montney Vs from Vp log in well 4. The depth scale is in mSSTVD.

**Fig. 7:**
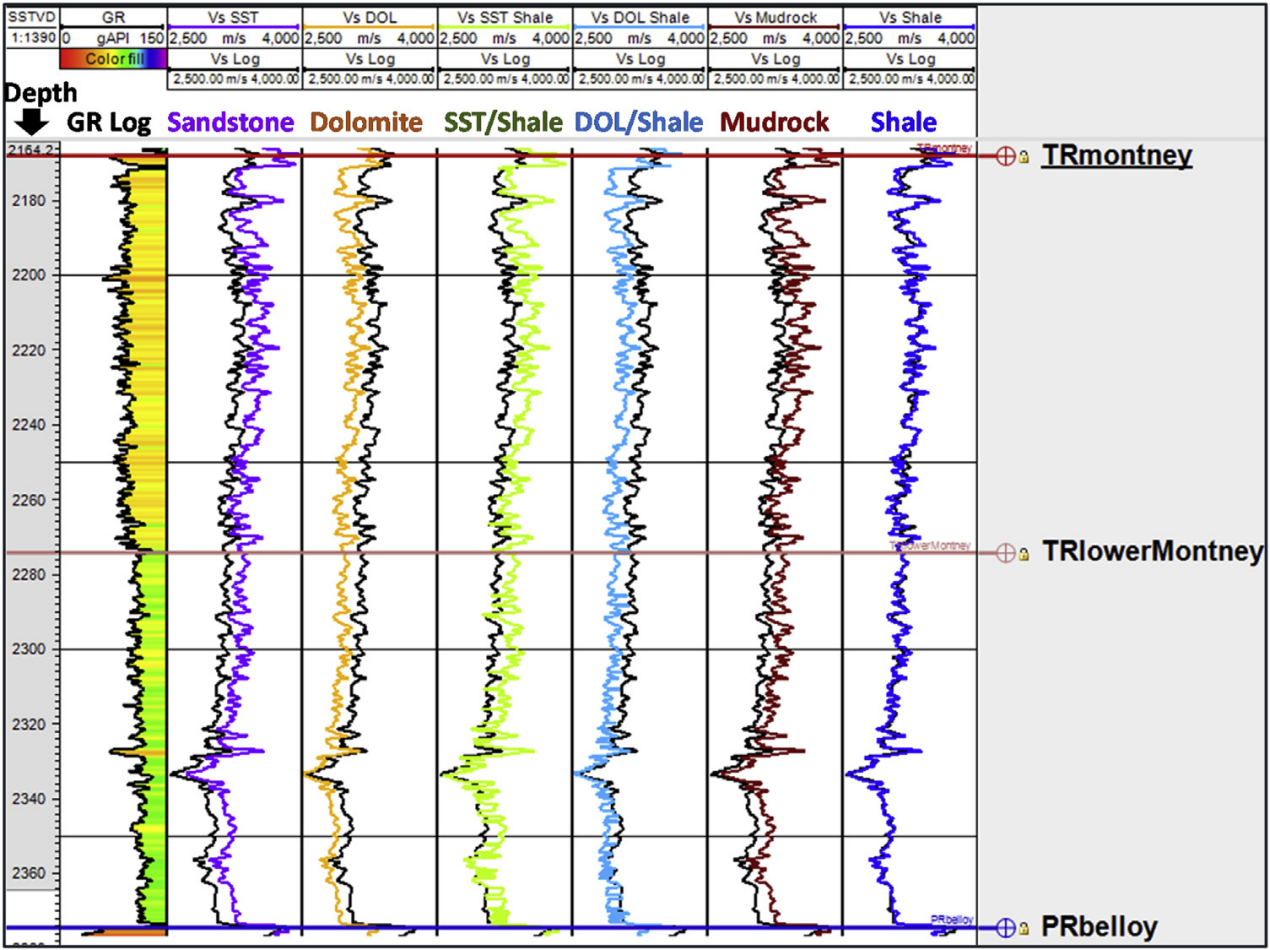
Lithology constant-based scenarios for estimating Montney Vs from Vp log in well 5. The depth scale is in mSSTVD.

**Fig. 8:**
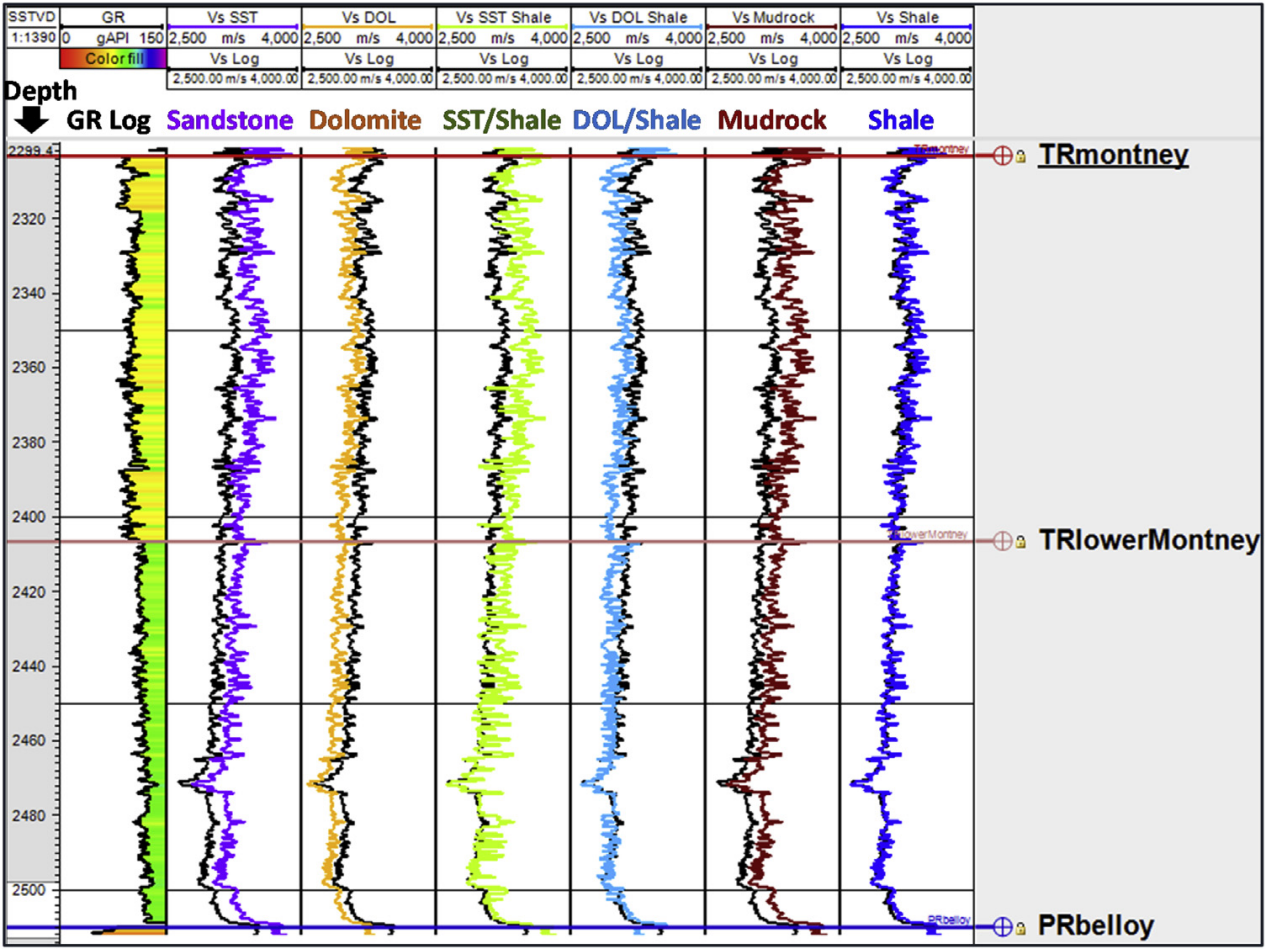
Lithology constant-based scenarios for estimating Montney Vs from Vp log in well 6. The depth scale is in mSSTVD.

Using the relations above, we generated six scenarios of Vs logs. In four of the scenarios, we hypothetically assumed that the Montney interval consists of a single lithology (Fig. 5, Fig. 6, Fig. 7, Fig. 8). In the remaining two scenarios, we performed the Vs estimation on the basis that the Montney interval is comprised of mixed lithologies of either sandstone and shale, or dolomite and shale. For the two mixed lithology scenarios tested, a shale lithology cut-off of 115° GR API [6] was defined from the GR log using shale limits measured from marine shale intervals (Fernie and Kaskapau Formations) overlying the Montney Formation in our study area. The six estimated Vs logs were compared with the measured Vs from dipole sonic logs (Fig. 5, Fig. 6, Fig. 7, Fig. 8). On the basis of regional knowledge, no limestone lithology constant scenario was tested.

A limitation of estimating Vs using Greenberg-Castagna [1] and Castagna et al. [5] lithology-based methods arises from the original lithology constants not being calibrated using data from our study area. These limitations necessitated our calibration of the estimated Vs logs using the measured Vs (which is also the validating Vs log) in the Montney Formation in our study area. Upon analyzing the outputs from the six Vs scenarios generated, we observed that in the Montney interval, the Vs SH estimated log (Fig. 5, Fig. 6, Fig. 7, Fig. 8, log track 1 from the right) had the best match with the measured Vs from the dipole sonic log (Fig. 5, Fig. 6, Fig. 7, Fig. 8), thus indicating that for the Montney Formation in our study area, the Greenberg-Castagna [1] shale lithology constants can be empirically used as a proxy Vs log estimator.

**Fig. 9:**
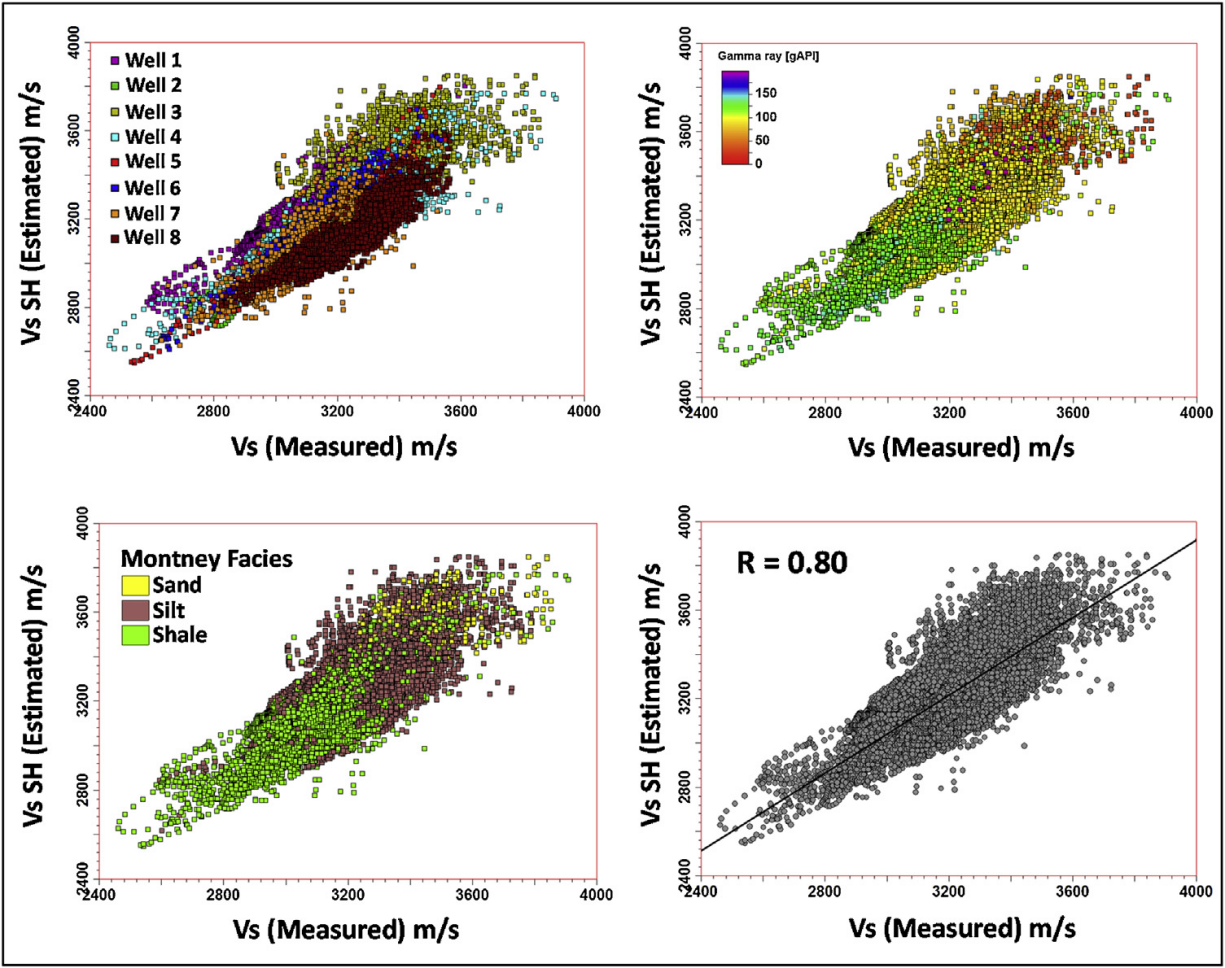
Montney empirically estimated Vs versus the measured Vs. This figure compares the estimated Vs SH with the Vs measured using the dipole sonic tool. The four plots depict the combined Vs SH versus measured Vs relationship for the eight wells studied. Each data point represents individual log values. The Montney facies shown are based on Montney GR-based lithofacies [6], where Sand: GR < 60 API, Silt: 60 ≤ GR ≤ 115 API, and Shale: GR > 115 API. The sands predominantly occur in the upper section of the Montney interval in the study area. The gross sand proportion estimated from well logs in the study area above the TRlower Montney marker is ˜2% [10]. The trend line in the bottom right plot is a linear regression line generated through the least squares method [27]. R is the correlation coefficient.

The cross plot in Fig. 9 shows the correlation between the estimated Vs SH logs and measured Vs logs from dipole sonic for the eight wells.

### Vs log estimation from multiwell Vp – Vs regression

**Fig. 10:**
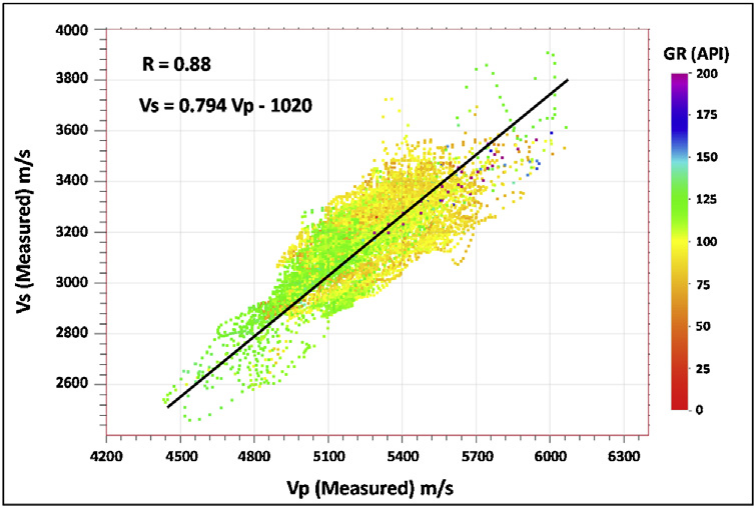
Montney multiwell regression dipole sonic Vp versus Vs relationship.

**Fig. 11:**
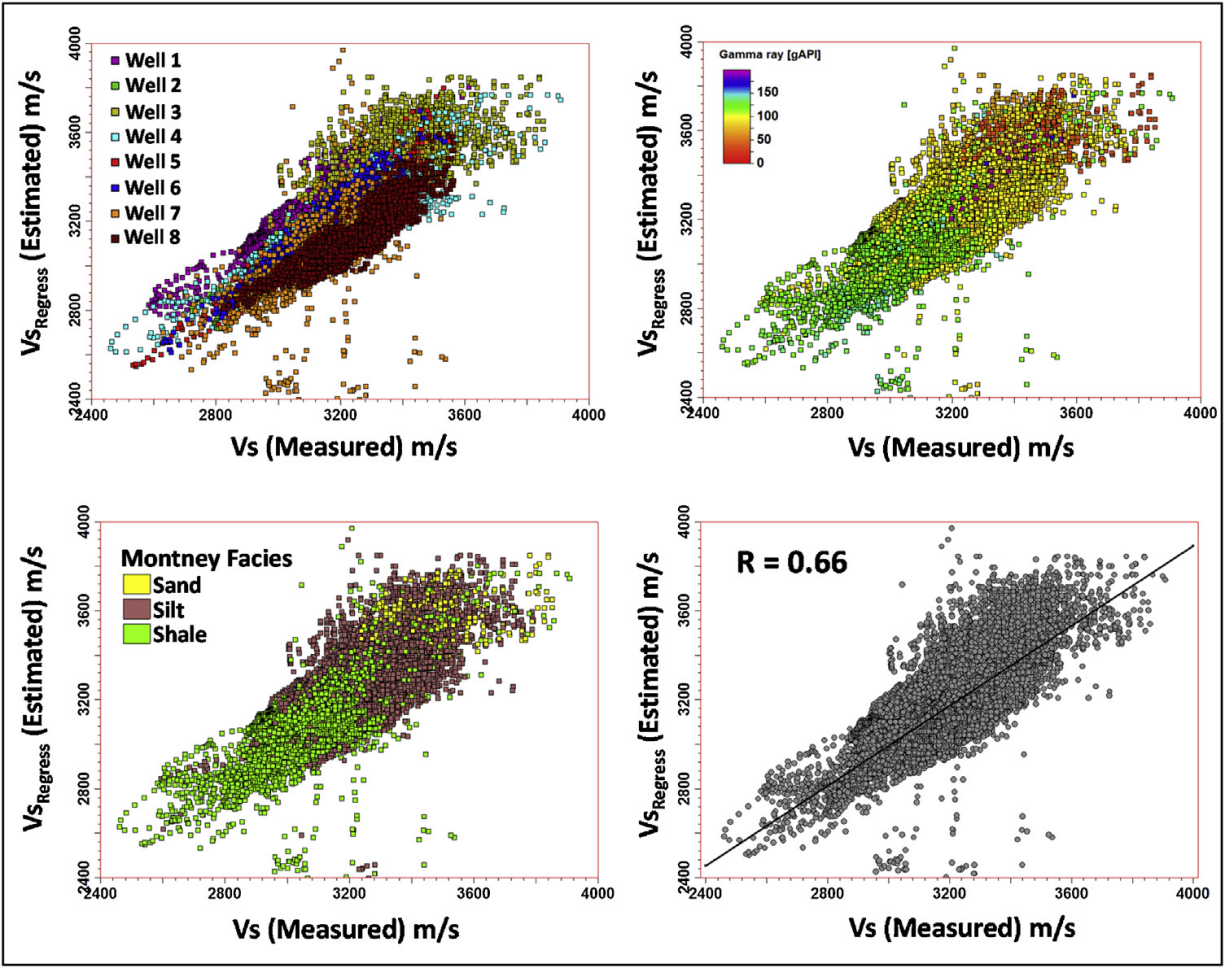
Montney multiwell regression-based Vs versus the measured Vs.

For the multiwell analysis, we performed a bivariate regression [25,26] using the dipole sonic Vp and Vs logs from the eight wells. The resulting regression equation (shown in Fig. 10) was used to estimate Vs logs in the eight wells. The estimated Vs (Vs_Regress_) was compared with the measured Vs from the dipole sonic tool, as shown in Fig. 11.

### Neural network estimation

Neural network estimation (NNE) techniques have been used for classifying variables and predicting objective functions in science and engineering disciplines for several decades [18,19,20]. In petroleum exploration and production, neural network methods [19] have seen an uptick in attention in recent years resulting from a rise in the adoption of machine learning techniques in geoscience workflows [23,24].

**Fig. 12:**
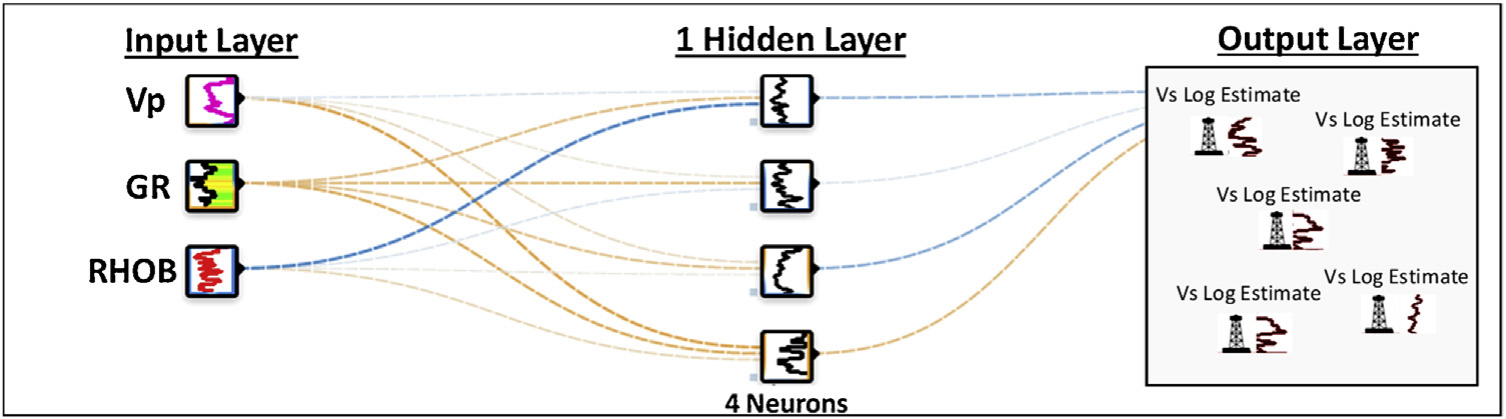
Schematic neural network setup for Vs log estimation in the Montney interval. In the output layer we show the use of the ANN model for estimating Vs logs at different well locations. Each square in the hidden layer represents the output from one neuron in the hidden layer. The neural network framework shown above was modified from playground.tensorflow.org.

In an artificial neural network approach, the variables (or neurons) in the input layer (Var_n1_…..Var_nx_) are trained to generate an estimate of the target or objective function (V_Tar_) [21]. The process involves the assignment of various weights to Var_n1_ – Var_nx_ in one or multiple hidden layers [22] (Fig. 12).

To test the NNE approach for estimating Vs logs from Vp logs, we implemented a feed-forward backward-propagating supervised artificial neural network (ANN) with an error minimizing function [22]. The ANN model was set up using the Vp log as input, complemented with the GR and RHOB logs (Fig. 12**)**. The ANN model comprised of one hidden layer, with the Vs log being the V_Tar_ (Fig. 12**)**.

**Fig. 13:**
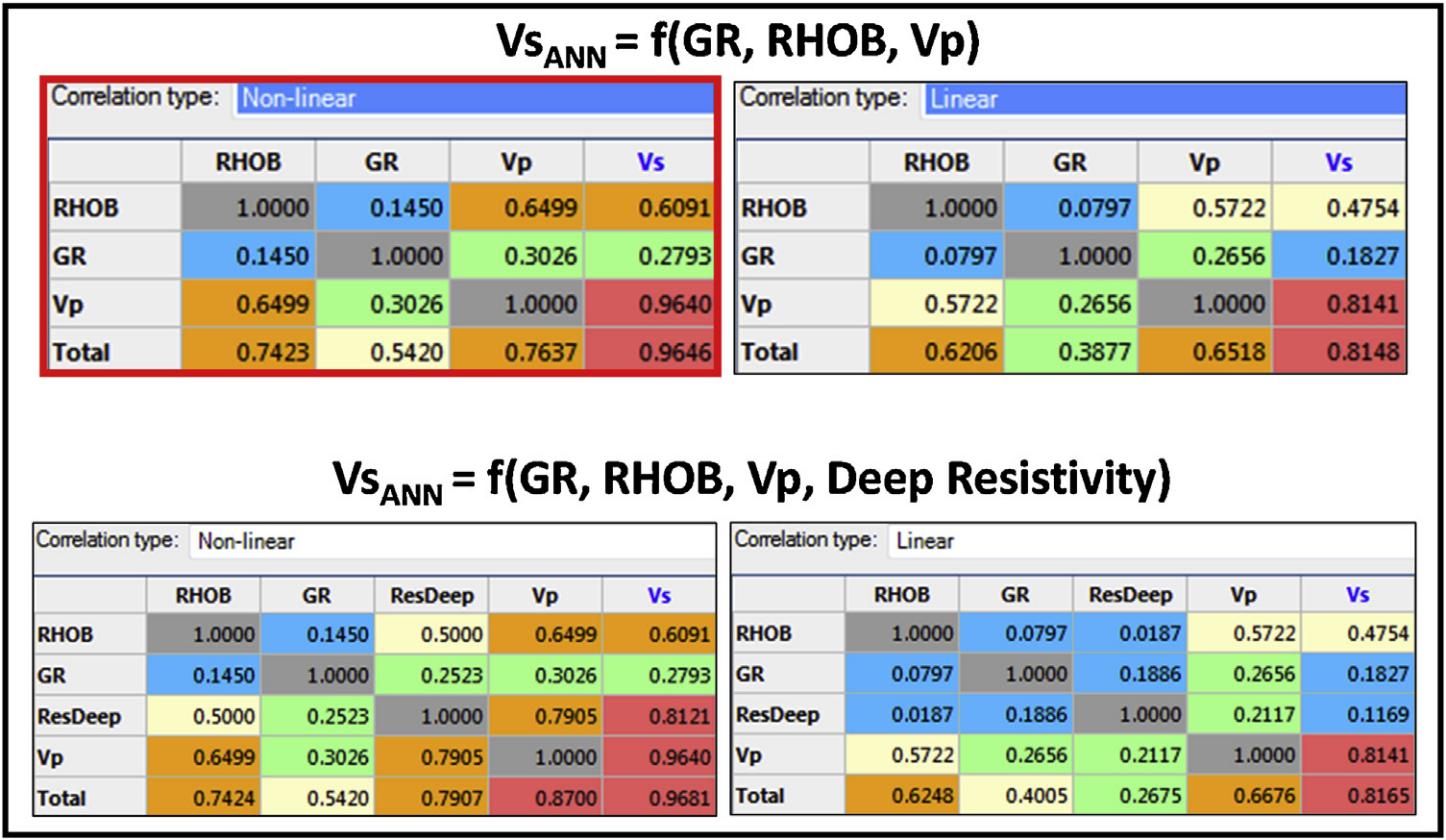
ANN model input logs correlation tables. The base case ANN model for this study was run using the non-linear correlation (the top left table insert) for Vs_ANN_ = f(GR, RHOB, Vp).

Three wells (1, 2, and 6) were used to train and test the model while Vs was estimated in the remaining five wells (3, 4, 5, 7, and 8) using the ANN model (Fig. 12, Fig. 13). An ANN model scenario was tested using a non-linear correlation between four well log inputs -Vp, GR, RHOB, and Deep Resistivity- (Fig. 13). However, this scenario yielded similar results as the three inputs used for the GR-RHOB-Vp-based ANN model (Table 1), likely due (in part) to the relatively weak correlation of the GR log to other input logs and the Vs log (the objective function).

**Table 1:** Montney Vs estimation ANN model characteristics.

Model Type	Input Logs	Number of Runs (Epochs)	Relative Error	Training Points	Testing Points
Base case	GR, RHOB, Vp	100	0.5533	55955	18651
Test scenario	GR, RHOB, Vp, Deep Resistivity	100	0.5560	55955	18651

The impact of the GR log’s weak correlation on the Vs_ANN_ estimation can be overcome by the replacing the GR log in the input layer with a log that has a higher correlation with other input logs and the V_Tar_. Alternatively, the ANN Vs estimation results can also be improved through deep-learning neural networks [7,18,22].

**Fig. 14:**
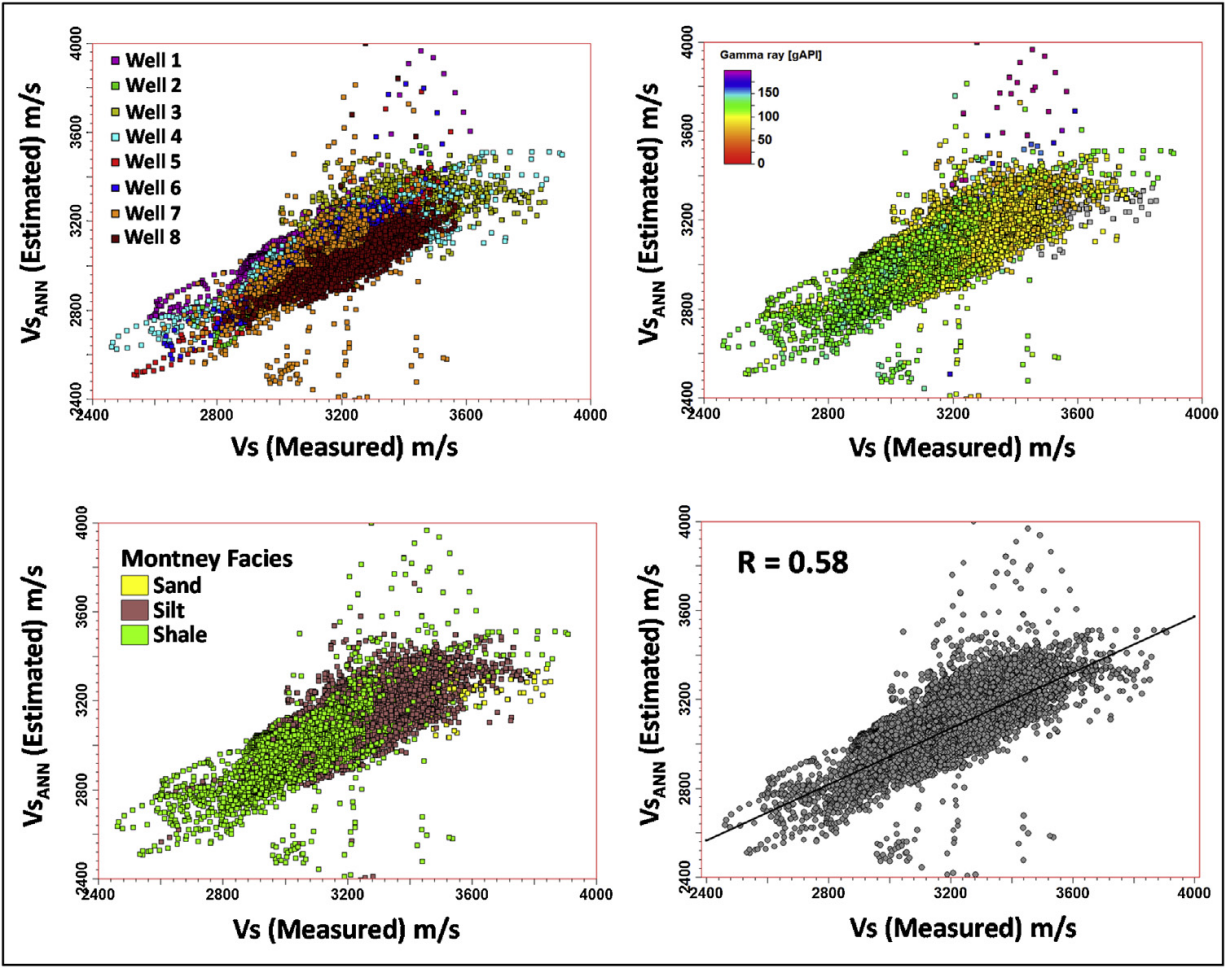
Montney ANN-estimated Vs versus the measured Vs.

As was performed for the earlier two Vs estimation approaches discussed, the Vs estimated from the ANN (Vs_ANN_) were compared with the measured Vs from the dipole sonic logging tool (Fig. 14).

A principal component analysis (PCA) of the Vs_ANN_ estimation model showed that the first two principal components (PC0 and PC1) captured almost 89% of the variations in the input dataset (Table 2). The greatest variations are shown by the direction of the first principal component.

**Table 2:** Montney Vs estimation ANN model principal components.

**Correlation Coefficients**	**PC0**	**PC1**	**PC2**
GR	0.8271	0.4246	0.3682
RHOB	−0.2926	0.9343	−0.2037
Vp	0.9131	−0.0853	−0.3988
Eigenvalue	1.6035	1.0605	0.3361
Contribution (%)	53.45	35.35	11.20
Cummulative Contribution (%)	53.45	88.80	100.00

**Fig. 15:**
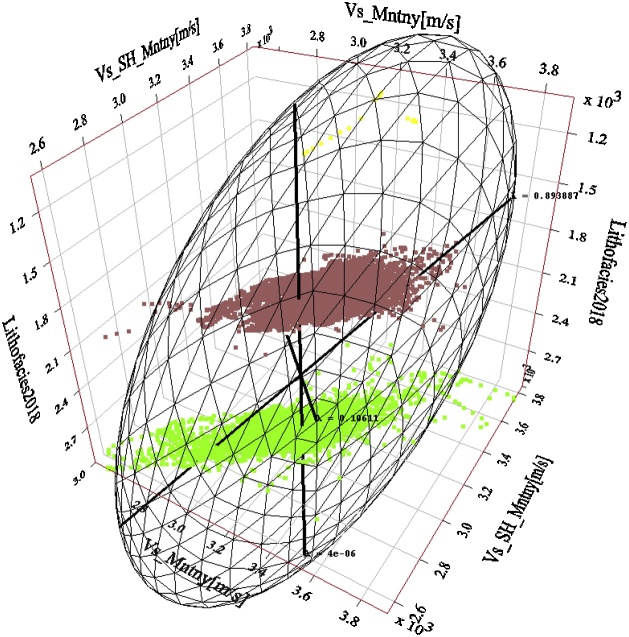
Principal components of the Montney Vs SH versus the measured Vs regression for wells 1–8. The estimated versus measured Vs regression was plotted using Montney facies as the third dimension. Two principal components capture essentially all of the variations in the dataset. The intersection of the three principal components is the mean Vs. The size of each principal component’s line segment is proportional to the standard deviation along the principal component. The mesh is a scaling-dependent representation of orthogonal directions of the dataset and does not preclude the points that lie outside the mesh from being included in the PCA. The direction of greatest variance occurs from the shale to silt facies, indicated by the principal component with a contribution (λ) of ˜0.894. Note that λ in this paper refers to the level of contribution of the principal component and not its eigenvalue. The Montney facies legend is the same as is shown in Figs. 9, 11 and 14.

**Fig. 16:**
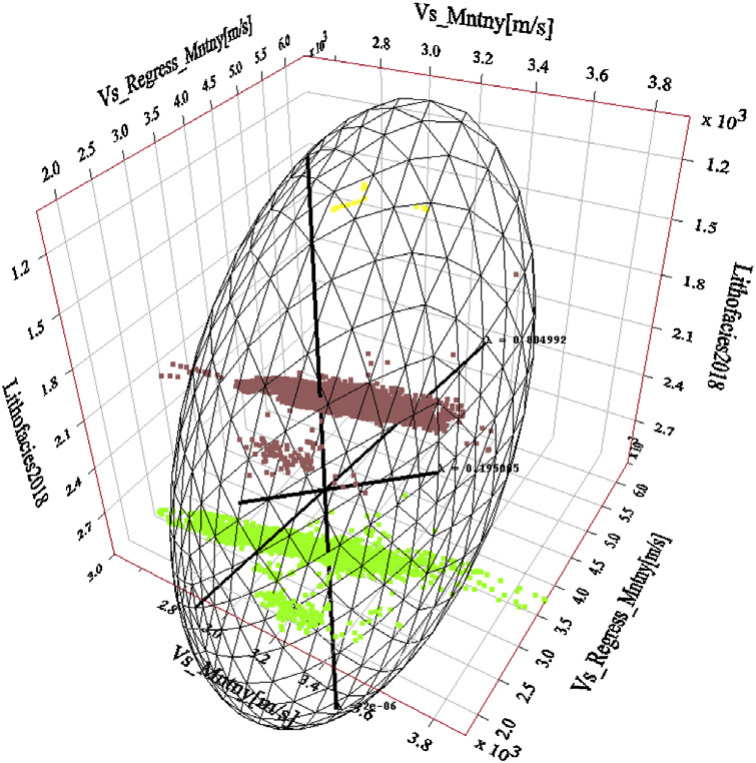
Principal components of the Montney Vs_Regress_ versus the measured Vs regression for wells 1–8. Two principal components also capture essentially all of the variations in the dataset, however, with a reduced λ of ˜0.805 for the first principal component. The direction of greatest variance appears to be driven by the variance in the shale facies rather than the variance in the silts facies. Both the silt and shale facies Vs estimates show a higher variance than the Vs SH estimate in Fig. 15. Principal components of the Montney Vs SH versus the measured Vs regression for wells 1–8. The estimated versus measured Vs regression was plotted using Montney facies as the third dimension. Two principal components capture essentially all of the variations in the dataset. The intersection of the three principal components is the mean Vs. The size of each principal component’s line segment is proportional to the standard deviation along the principal component. The mesh is a scaling-dependent representation of orthogonal directions of the dataset and does not preclude the points that lie outside the mesh from being included in the PCA. The direction of greatest variance occurs from the shale to silt facies, indicated by the principal component with a contribution (λ) of ˜0.894. Note that λ in this paper refers to the level of contribution of the principal component and not its eigenvalue. The Montney facies legend is the same as is shown in Figs. 9, 11 and 14.

**Fig. 17:**
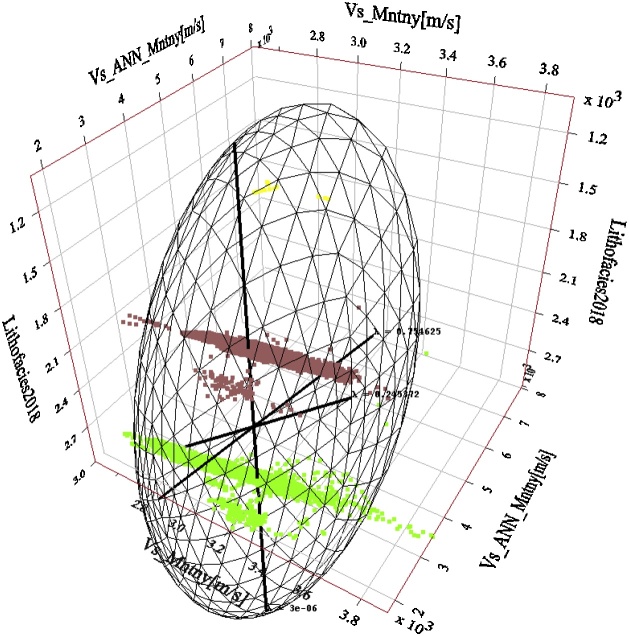
Principal components of the Montney Vs_ANN_ versus the measured Vs regression for wells 1–8. The relative importance of the first principal component is further reduced with a λ of ˜0.755. The first and second principal components still essentially capture all of the variations in the dataset. The direction of greatest variance appears to still be driven by the variance in the shale facies rather than the variance in the silts facies. Both the silt and shale facies show a smaller variance in the Vs_ANN_ estimate. The facies legend is the same as is shown in Figs. 9, 11 and 14.

Well by well PCA analysis of the estimated Vs to the measured Vs regression (performed for the closest matching Vs estimation approaches so far presented in this paper, i.e. Vs SH, Vs_Regress_) confirms the predominance of PC0 and PC1. This finding lends credence to the use of Vs SH and Vs_Regress_ approaches for estimating dynamic Vs in the Montney Formation in the study area. Fig. 15, Fig. 16, Fig. 17 show the results of the PCA for the Vs SH, Vs_Regress_, and Vs_ANN_ estimations, compared with the measured Vs log.

**Fig. 18:**
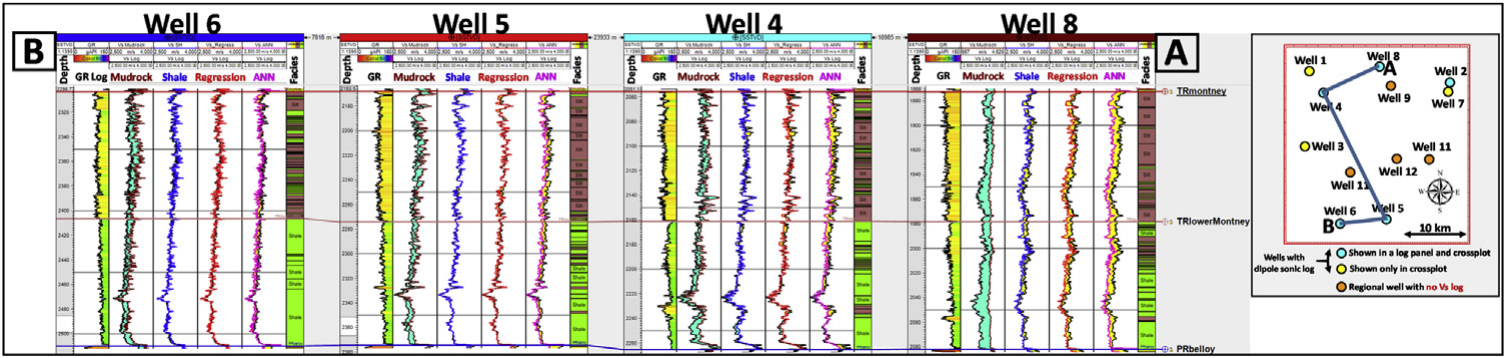
Well correlation comparison of the Montney Vs estimation results with the measured Vs log. The measured Vs log is in the black curve in all the log tracks. A turquoise fill means that the estimated Vs log value is higher than the measured Vs. A yellow fill means that the value of the measured Vs is higher than the estimated Vs log. The facies legend is the same as is shown in Figs. 9, 11 and 14.

As can be seen from Fig. 18, among the Vs estimation approaches evaluated so far, the ANN method yielded the most subpar results. An option of increasing the number of input logs used in the ANN model to improve its performance exists, however, the number of principal components that would be generated by the ANN model to explain variations in the dataset would also increase. From the principal component analyses that were performed on a well by well basis, in all eight wells, mainly two principal components were sufficient to capture the variance between the estimated Vs logs and the measured Vs logs.

As an alternative to using the earlier suggested deep-learning neural network techniques [7, 18, 22] or an increased number of input logs to improve the NNE, more well logs can be examined using correlation tables and principal component analysis (Fig. 13 and Table 2) to identify a replacement log for the GR with better correlation to other logs used in the ANN estimation model.

### Testing for the effects of fluid and clay content on Montney velocity

Compressional velocity is sensitive to variations in Ø, and fluids [8,9]. Vs is sensitive to fluid type, however, with lesser sensitivity than Vp [9]. The Montney interval is predominantly gas bearing in our study area, with the main production focus being gas condensates produced from multi-stage hydraulically fractured horizontal wells. The upper portion of the Montney interval is primarily siltstone-dominated in the study area (Fig. 18), with increasing shaly-siltstone/shale lithology towards the basal portion of the reservoir [10] (Fig. 18).

For gas reservoirs, Marion & Jizba [11] investigated the influence of Ø and clay content on Vp and Vs. They proposed that Vp and Vs can be estimated by taking into account the matrix Ø and V*_sh_* using the following relations:

(8) Vp=4.82-5.04ϕ-0.597 Vsh

(9) Vs=3.26-3.03ϕ-0.892 Vsh

Where: Vp is Compressional velocity Vs (m/s), Vs is Shear velocity Vs (m/s), V_sh_ = Clay volume (fraction), and Ø is Rock porosity (fraction).

An advantage of the Marion & Jizba technique [11] is its usefulness in estimating Vs without relying on the Vp log. We tested their approach using three estimation scenarios which considered three of the common V*_sh_* correction techniques documented in the literature [[12], [13], [14]]. The following relations were used for the three estimations:

(10) $$Vs\;MJ\;Larionov=3.26-3.03\phi -0.892V_{sh,\;Larionov}$$

(11) $$Vs\;MJ\;Clavier=3.26-3.03\phi -0.892V_{sh,\;Clavier}$$

(12) $$Vs\;MJ\;Stieber=3.26-3.03\phi -0.892V_{sh,\;Stieber}$$

Where Vs MJ Larionov, Clavier, and Stieber correspond to the resulting Vs logs that take into account the V_sh_ corrections expressed as:

(13) $$V_{sh,\;Larionov}=0.08(2^{3.7V_{sh,\;Linear}})$$

(14) $$V_{sh,\;Clavier}=1.7-{\left[3.38-\left(V_{sh,\;Linear}-0.7\right)\right]}^{0.5}$$

(15) $$V_{sh,\;Stieber}=\frac{V_{sh,\;Linear}\;}{3-2V_{sh,\;Linear}\;}$$

(16) $$V_{sh,\;Linear}=\frac{GR\;-{\;GR}_{sand}\;}{{\;GR}_{shale}\;-{\;GR}_{sand}\;}$$

The GR*_sand_* and GR*_shale_* cut-offs were defined at 60*o* GR API and 115*o* GR API respectively, according to [6]. The input Ø was determined from the bulk density (RHOB) by taking into account the matrix and fluid densities as described in [6].

**Fig. 19:**
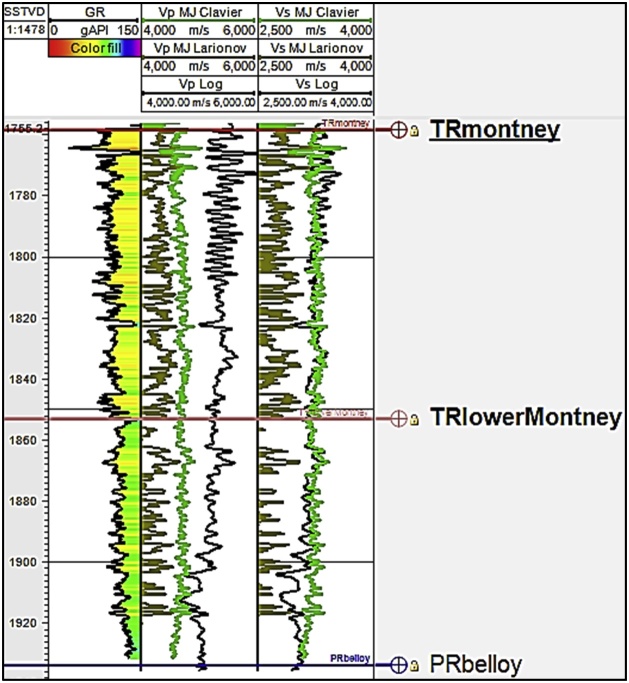
Montney Vp and Vs estimated logs using Marion & Jizba [11] relations. The measured Vp and Vs from the dipole sonic tool are shown as black curves in tracks 1 and 2 from the right. This is an example from well 2.

Using well 2 (Figs. 3, 5, and 18) to demonstrate our test results from the three Marion & Jizba [11] Vs estimation scenarios, we show that the Vs MJ Stieber approach yields Vs log values below 2500 m/s in the Montney interval which generally underestimates the Vs in the formation (Fig. 10, Fig. 19). Although the Vs MJ Larionov yielded values that were generally greater than 2500 m/s, the Vs MJ Larionov also underestimates the Vs in the formation (Fig. 10, Fig. 19). The Vs MJ Clavier had the closest approximation to the measured Vs from the dipole sonic (Fig. 19). However, the Vs MJ Clavier match falls short of the best match we earlier observed from the Greenberg-Castagna [1] Vs SH estimation (Fig. 5, Fig. 6, Fig. 7, Fig. 8, Fig. 9).

For completeness, as a part of testing the Marion & Jizba [11] approach, we also tested the Vp estimation (Eq. (8)) using the three V_sh_ correction scenarios to assess how the Vp estimated using their relation compared with measured Vp logs. Similar to our observations for the Vs estimates (also using well 2 as an example), we found that the Vp MJ Stieber (which was consistently less than 4000 m/s) also underestimated the Vp in the formation (not shown). The Vp MJ Larionov showed a greater Vp underestimation compared to the Vp MJ Clavier result (Fig. 19). A possible source of the Vp and Vs mismatches observed using the Marion & Jizba [11] approach may be linked in part to the shaly sandstone lithology upon which the authors’ analysis was performed.

An investigation of the influence of Ø and fluid compositional variation on Vp and Vs in the Montney Formation has been performed in the Kaybob area (located east of our study area) by Oraghalum et al. [7]. As the results from the Marion & Jizba [11] approach yielded Vs estimations that were subpar compared to the earlier estimated Vs logs (especially the Vs SH and Vs_Regress)_, no further evaluation of the Marion & Jizba method was performed.

## Summary and conclusions

**Fig. 20:**
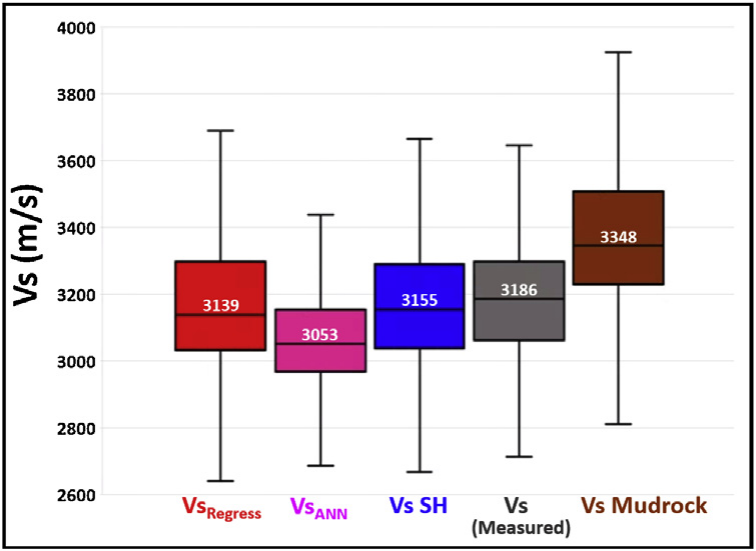
Comparison of the estimated Montney Vs (in 8 wells) with the measured Vs from dipole sonic.

By testing various theoretical methods for estimating dynamic Vs from Vp logs, we have shown that in the study area, the use of the Greenberg-Castagna shale lithology relation [1] yielded the closest Vs log estimates compared to the measured Vs log (Figs. 5–8, 18, and 20).

The multiwell bivariate regression using the dipole sonic Vp and Vs logs yielded the next-best estimates (Fig. 18, Fig. 20). This approach can be used as a complementary method for Vs log estimation to assess the level of uncertainty on the Vs logs estimated in the study area.

The availability of Vs logs from the dipole sonic tool in the wells 1–8 aided in validating the predictive capability of the dynamic Vs estimation techniques assessed in this study.

Given that the Montney Formation is siltstone-dominated in our study area, the match between the Vs SH estimation approach and the measured Vs log demonstrates that the Vs SH empirical relation can representatively capture the Vs in the Montney interval. By virtue of the form of the Vs SH equation, it can be seen that the Vs SH relation is similar to the Vs_Regress_ equation that was based on the multiwell regression of dipole sonic Vp and Vs logs (Eq. (6) and Fig. 10).

Without performing the assessment presented above, the seemingly logical relation for Vs log estimation from the Vp log (using empirical relations) in the siltstone-dominated Montney interval could have been the mudrock lithology constant-based Castagna et al. relation [5]. However, as we have shown, the Vs estimated using the mudrock lithology constants yielded sub-optimal Vs estimates compared to the Vs log estimated using the Greenberg-Castagna [1] shale lithology constants (Fig. 18, Fig. 20). Also, other pure lithology (sandstone and dolomite) and mixed lithology scenarios tested using the Greenberg-Castagna relation [1] yielded sub-par Vs log estimates compared to the Vs SH log, as earlier shown.

The Vs SH log estimation method presented in this paper, therefore, demonstrates a relatively more efficient way of estimating Vs logs from Vp logs in the Montney Formation, in the study area. This multi-technique assessment approach can be performed in other areas of the Montney Formation or other shale and tight reservoirs to determine the most optimal Vs estimation technique from Vp or other complementary well logs. Validation with measured Vs logs must be an essential component of any approach that is adopted.

## Sources of funding

This work was partially funded by the Natural Science and Engineering Research Council Collaborative Research and Development (NSERC CRD) Grant - Project #J452752 - 13 to Clarkson.
